# Editorial: Targeting glioblastoma: Mechanisms of pathology and novel therapeutic strategies

**DOI:** 10.3389/fmolb.2023.1181398

**Published:** 2023-03-27

**Authors:** Magdalena Kusaczuk, Xin Zhang

**Affiliations:** ^1^ Medical University of Bialystok, Bialystok, Poland; ^2^ Jiangmen Central Hospital, Jiangmen, China

**Keywords:** glioblastoma, gene signature, immunity, prognosis, risk score, overall survival

Glioblastoma (GBM) is considered one of the most abundant types of glial tumors, with poor prognosis of survival. Although numerous advances have been made in understanding the pathophysiology of GBM and the development of novel therapeutic regimens, little improvement in the median survival rate of GBM patients has been achieved (Oronsky et al., 2021). This highlights the urgent need for more effective therapeutic strategies and identification of specific prognostic biomarkers for optimization of clinical management of this neoplasm. The development of such therapies depends not only on the identification of genes and molecules controlling GBM growth, but also on the recognition of changes in the immune system responsible for progression of the disease. Although a large number of molecular biomarkers have been employed for the pathological diagnosis and prognostic evaluation of GBM (e.g., IDH, EGFR, p53), novel prognostic predictors are constantly quested to improve patients’ outcome (Ludwig and Kornblum, 2017; Oronsky et al., 2021). Consequently, drugs targeted at specific molecular biomarkers of GBM are also a subject of extensive clinical investigations, however most of these ultimately fail due to the high invasiveness and drug resistance of GBM. As such, characterization of novel and accurate biomarkers and their therapeutic aims seems essential not only for GBM diagnosis and prognosis prediction, but also for drug screening (Yang et al., 2022).

Within this Research Topic we have collected contributions focused on advances in genetic predisposition and novel biomarkers linked to GBM, in order to shed more light to the complicated nature of this malignancy.

As such, Jin et al. used an integrative genomic approach to characterize a Chinese GBM cohort in order to search for potential prognostic biomarkers. They assembled a dataset comprised of RNA-seq, whole-exome sequencing, and clinical data from 46 patients with primary malignant glioma. Using patients’ RNA-seq data Authors divided patients into a GBM group or a low-grade glioma (LGG) group. Gene set enrichment analysis comparing LGG vs. GBM group suggested that GBM showed upregulation of multiple pathways related to genome integrity and immune cell infiltration. Furthermore, the comparison of somatic mutations between the two groups revealed mutation in *TOPAZ1* gene as a novel mutation associated with GBM. Comparison between GBM patients with good and poor survival prognosis revealed six significantly mutated genes (*TRIML2*, *PKD1*, *ROCK1*, *OBSCN*, *ADCY7*, and *HECTD4*) and two genes with more copy number mutations alterations (*NTRK1* and *B2M*) within the poor prognosis group. Collectively, this genomic and transcriptomic data revealed that GBM patients showed distinct characteristics related to chromosome integrity, individual genes, and infiltrating immune cells compared to LGG group, suggesting potential prognostic factors in this cohort of patients.

Likewise, Tong et al. aimed at discovering the hub genes of glioma and investigating their prognostic and diagnostic usefulness. In their study, Authors collected the mRNA expression profiles and clinical information from glioma patients in the TCGA, GTEx, GSE68848, and GSE4920 databases. They identified the 17-gene signature, for which the risk score accurately predicted overall survival in glioma patients and could be considered an independent prognostic model. Further bioinformatics analysis revealed 7 hub genes with great drug targeting potential. Out of these hub genes, the ribosomal protein L39 (RPL39), was found to be closely related to the prognosis of glioma patients. Of note, overexpression of RPL39 *in vitro* markedly enhanced the proliferation and migration of GBM cells, whereas knockdown of RPL39 resulted in opposite effects. Altogether, this research identified a novel 17-gene signature contributing to maximization of the prognostic assessment and molecular targeted therapy of glioma.

Moreover, Deng et al. analyzed the B7-CD28 family gene expression profiles, and developed a B7-CD28 family-based prognostic signature to predict survival and immune host status in diffuse gliomas. Based on the bioinformatics analysis Deng et al. developed a B7-CD28 family-based signature that consisted of *CD274*, *CD276*, *PDCD1LG2* and *CD80* genes. This gene signature has been shown to have significant prognostic value, and serve as a biomarker distinguishing pathological grade and IDH mutation status in diffuse gliomas. Furthermore, Authors found this gene signature to correlate with intensity of immune response and immune cell population, as well as several other important immune checkpoint genes, holding a great potential to be a predictive immune marker for immunotherapy and tumor microenvironment. Finally, a B7-CD28 family-based nomogram was established to predict patients’ life expectancy, providing novel insights into immunotherapy of diffuse glioma.

Finally, Wei et al. explored the expression profile of the RNA-binding motif protein 8A (RBM8A) as important prognostic marker and therapeutic target in GBM. They analyzed genes, genetic mutations, methylation modifications and molecular functions associated with alterations in RBM8A expression bioinformatics approach. They discovered that RBM8A was significantly overexpressed in GBM in comparison to control samples and its level correlated with tumor purity. They also identified 13 mRNAs that were associated with overall survival of GBM patients, and found RBM8A to compete with DLEU1 for binding to miR-128-1-5p, while connecting aberrant RBM8A expression with tumor infiltration by immune cells. Overall, this study linked RBM8A expression to GBM pathobiology and patients’ prognosis making this protein a potential candidate for therapeutic target against this disease.

All of the collected papers support the notion that identification of molecular and genetic risk factors linked to GBM may contribute to more precise diagnosis and the development of more efficient targeted therapeutics against this tumor.
